# Tunable microwave metasurfaces for high-performance operations: dispersion compensation and dynamical switch

**DOI:** 10.1038/srep38255

**Published:** 2016-11-30

**Authors:** He-Xiu Xu, Shiwei Tang, Shaojie Ma, Weijie Luo, Tong Cai, Shulin Sun, Qiong He, Lei Zhou

**Affiliations:** 1 State Key Laboratory of Surface Physics, Key Laboratory of Micro and Nano Photonic Structures (Ministry of Education) and Physics Department, Fudan University, Shanghai 200433, China; 2 Air and Missile Defense College, Air force Engineering University, Xi’an, 710051, China; 3 Department of Physics, Faculty of Science, Ningbo University, Ningbo 315211, China; 4 Shanghai Engineering Research Center of Ultra-Precision Optical Manufacturing, Green Photonics and Department of Optical Science and Engineering, Fudan University, Shanghai 200433, China; 5 Collaborative Innovation Center of Advanced Microstructures, Fudan University, Shanghai 200433, China

## Abstract

Controlling the phase distributions on metasurfaces leads to fascinating effects such as anomalous light refraction/reflection, flat-lens focusing, and optics-vortex generation. However, metasurfaces realized so far largely reply on *passive resonant* meta-atoms, whose *intrinsic dispersions* limit such *passive* meta-devices’ performances at frequencies other than the target one. Here, based on *tunable* meta-atoms with varactor diodes involved, we establish a scheme to resolve these issues for microwave metasurfaces, in which the dispersive response of each meta-atom is precisely controlled by an external voltage imparted on the diode. We experimentally demonstrate two effects utilizing our scheme. First, we show that a *tunable* gradient metasurface exhibits *single-mode high-efficiency* operation within a wide frequency band, while its passive counterpart only works at a single frequency but exhibits deteriorated performances at other frequencies. Second, we demonstrate that the functionality of our metasurface can be dynamically switched from a specular reflector to a surface-wave convertor. Our approach paves the road to achieve dispersion-corrected and switchable manipulations of electromagnetic waves.

Metasurfaces^1^,^2^,^3^,^4^, planar metamaterials with tailored reflection/transmission phase profiles, attracted enormous interest recently^5^,^6^,^7^,^8^. Many fascinating effects were discovered based on metasurfaces in different frequency domains, such as anomalous light refraction/reflection^9^,^10^,^11^,^12^,^13^,^14^,^15^,^16^,^17^, surface wave (SW) couplers^2^,^18^, vortex beam generation^19^, flat lens^20^,^21^,^22^,^23^,^24^,^25^,^26^,^27^,^28^, polarization control^29^,^30^, holograms^31^,^32^,^33^,^34^, and photonic spin-Hall effect^35^,^36^. To design a metasurface with certain functionality, one typically combines a set of resonant meta-atoms with distinct electromagnetic (EM) responses to form a planar structure, such that the whole device exhibits the desired profiles of transmission/reflection amplitude/phase. Such a strategy has been successfully implemented in the past years to design various metasurfaces working for different purposes^1^,^2^,^3^,^4^,^5^,^6^,^7^,^8^,^9^,^10^,^11^,^12^,^13^,^14^,^15^,^16^,^17^,^18^,^19^,^20^,^21^,^22^,^23^,^24^,^25^,^26^,^27^,^28^,^29^,^30^,^31^,^32^,^33^,^34^,^35^,^36^.

However, such a designing strategy only works for a *single* frequency, since the desired phase profile cannot maintain at other frequencies due to the intrinsic dispersions of passive resonant meta-atoms. Figure 1 schematically illustrates the underlying physics based on a reflective metasurface requiring a linear reflection-phase profile. Suppose a collection of meta-atoms have been chosen to form a device exhibiting an ideal linear phase profile at the frequency *f*_0_ (red line in Fig. 1a), such a *strict* linear relationship cannot be satisfied at other frequencies (blue lines) with the same device, since all resonant meta-atoms exhibit Lorenz-type phase dispersions (inset to Fig. 1a) and thus the phase difference between adjacent meta-atoms varies as a function of frequency. In particular, the phase gradient must decrease to zero at frequencies far away from *f*_0_ where all resonant behaviors die off. As a result, while at *f*_0_ the device can support single-mode anomalous reflection dictated by the generalized Snell’s law (Fig. 1e), at other frequencies, the device’s performance is significantly decreased with undesired modes appearing and deteriorated working efficiency (Fig. 1c). Such an issue seems inherent to all metasurfaces based on *passive* resonant meta-atoms, working in both reflection^2^,^11^,^12^,^13^,^27^,^28^,^29^,^33^,^34^,^35^ and transmission^1^,^4^,^8^,^9^,^10^,^14^,^15^,^16^,^17^,^18^,^19^,^20^,^21^,^22^,^23^,^24^,^25^,^26^,^30^,^31^,^32^,^36^ geometries and relying on either phase or amplitude modulations^6^,^7^. Although the phase profiles in geometric-phase-based metasurfaces can immune from the frequency change, the real performances of such devices are dictated by the EM responses of their basic meta-atoms, which are still strongly frequency dependent^20^,^34^,^35^,^36^. We emphasize that such “*chromatic aberrations*” cannot be solved by simply expanding the working bandwidths of metasurfaces^11^,^12^,^13^,^14^,^15^,^16^ using low-Q and/or multi-mode resonators, since the frequency dispersion is intrinsic to all passive resonators.

In this paper, we *experimentally* demonstrate that such a limitation can be overcome by making *tunable* metasurfaces with meta-atoms controlled by external knobs (see inset to Fig. 1b). Borrowing similar *technical* ideas from previous studies on tunable reflectarrays/transmitarrays^37^,^38^,^39^,^40^,^41^ to involve varactor diodes into the meta-atom structures, we can individually control the reflection phase of each active unit by applying an appropriate voltage on the diode, so that the distorted phase profiles of the passive metasurface at off-working frequencies (*f* ≠ *f*_0_) can be rectified (see Fig. 1b). Consequently, our tunable metasurface can not only work well at the target frequency (Fig. 1f), but also works in the entire frequency band (Fig. 1d) with single-mode operation and high efficiency. We further experimentally demonstrate that the functionality of our device can be dynamically switched from a conventional reflector to a surface wave (SW) coupler, again based on the tunable and precise local-phase control. Such switchable functionality, not realized in previous SW couplers^2^,^18^, can be very useful in stealth applications where both normal detection and radar cross-section reduction are required. Our “tunable” approach, distinct from available strategies confined to homogenous metasurfaces and/or gradient-index materials with fabrication complexity and limited working frequencies^42^,^43^,^44^,^45^,^46^,^47^,^48^,^49^,^50^,^51^,^52^,^53^,^54^,^55^, opens the door to achieve dynamical control on the dispersions and functionalities of *inhomogeneous* metasurfaces, leading to many other exciting new applications such as aberration-free or functionality-switchable meta-lenses and tunable meta-devices including meta-holograms, flat sensors, etc.

## Results

### Dispersion issues in passive metasurfaces

**Figure 2:**
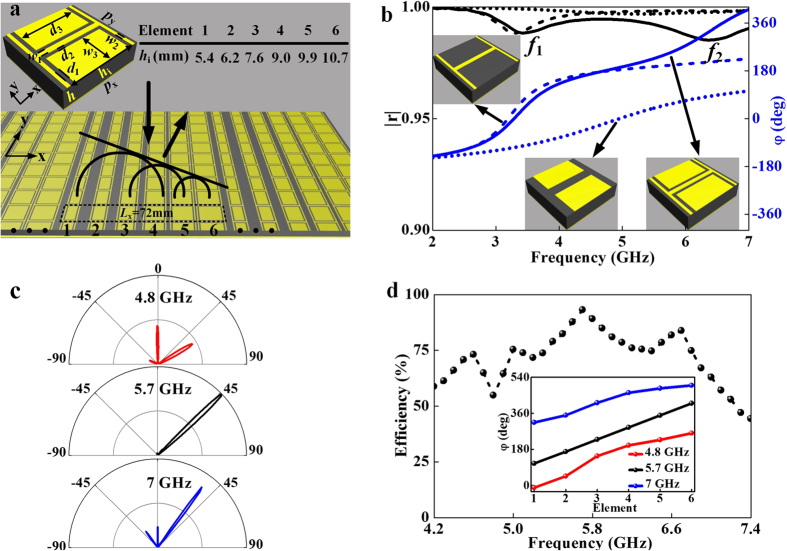
Dispersion-induced functionality deteriorations in a typical passive metasurface. (**a**) Geometry of the basic meta-atom and a gradient metasurface composed by six meta-atoms with parameter *h*_*i*_ varying from 5.4 mm to 10.7 mm. (**b**) FDTD-simulated spectra of reflection amplitude (left axis) and phase (right axis) of three periodic metasurfaces with unit-cells being the second meta-atom as shown in the inset to (**a**) (solid lines), meta-atom with patch only (dotted lines), and meta-atom with “H” only (dashed lines). (**c**) FDTD simulated far-field scattering patterns of the gradient metasurface at three typical frequencies. (**d**) Calculated anomalous-reflection efficiency of the gradient metasurface as a function of frequency. The efficiency was defined as the ratio between anomalously reflected power 
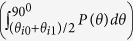
 with *θ*_*i*0_ and *θ*_*i*1_ being the reflection angles of normal and anomalous modes, respectively) and the totally reflected power 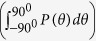, calculated by integrating the scattered-field intensity based on the FDTD-simulated patterns. Inset depicts the phase profiles at three selected frequencies. Geometrical parameters of the meta-atoms: *p*_x_ = *p*_y_ = 12 mm, *w*_1_ = 0.8 mm, *w*_2_ = 0.5 mm, *w*_3_ = 5.1 mm, *d*_1_ = 0.25 mm, *d*_2_ = 0.5 mm. The spacer layer is assumed as the F4B dielectric board with *ε*_r_ = 2.65, *h* = 6 mm, tanδ = 0.001, and all metallic films are assumed of 36 μm thickness.

To understand the dispersion issue in passive metasurfaces and lay a basis for designing our tunable structures, we first design a passive metasurface and analyze its wave-manipulation properties. Our meta-atom is a tri-layer structure, consisting of a composite planar resonator coupled with a metallic ground plane through a dielectric spacer (inset to Fig. 2a). To enlarge the working bandwidth of our device, we purposely design the meta-atom to contain two resonant modes, dictated by the metallic “H” structure and the metallic patch, respectively (Fig. 2a). The couplings between these planar structures and the ground plane generate two magnetic resonances, evidenced by the dips in the *x*-polarized reflectance spectra calculated by finite-difference-time-domain (FDTD) simulations, for two different systems with either only “H” or patch retained in the top layers (dashed and dotted lines in Fig. 2b). Detailed circuit-model analyses on these two modes can be found in Supplementary Information. With both “H” and patch present, our meta-atom can possess a much enlarged working bandwidth (~2–7 GHz) due to the coupling of two resonances (solid line in Fig. 2b). However, we will show that such bandwidth-enlargement *cannot* really improve the working performances of our metasurfaces, due to the intrinsic frequency dispersions of the passive resonant units.

We now design a gradient reflective metasurface using the basic meta-atom structure. Set the target frequency at *f*_0_ = 5.7 GHz, we select six meta-atoms via carefully adjusting their structural details such that they exhibit the desired reflection phases following the relationship *φ*(*x*) ∼ *ξ*(*x*) with *ξ* ≈ 0.74*k*_0_ at this very frequency. Since the absorptions are generally weak due to low resonance quality factors of these structures (see Fig. 2b), we purposely adjusted the structures of six meta-atoms such that their reflection amplitudes are all nearly 1 (with fluctuations less than 0.1). The amplitude fluctuations over meta-atoms thus have much less pronounced effect on the device’s working efficiency than the corresponding phase variations. The six meta-atoms form a supercell of our passive metasurface, as shown in Fig. 2a. We then employed FDTD simulations to study the scattering patterns of such metasurface illuminated by *x*-polarized EM waves, at different frequencies. While the scattering pattern at *f*_0_ = 5.7 GHz (middle panel in Fig. 2c) does exhibit a single-mode reflection at the designed angle (*θ*_r_ ≈ 47°) with diffractions to other channels well suppressed, multi-mode diffractions are significant at other frequencies (upper and lower panels in Fig. 2c). As a result, the true working efficiency of our device drops dramatically as frequency leaves 5.7 GHz, as shown in Fig. 2d. The physics can be understood from the inset to Fig. 2d, where the phase profiles of our device are depicted at three typical frequencies. Whereas a prefect linear profile is found at 5.7 GHz, *φ*(*x*) are obviously distorted at 4.8 GHz and 7 GHz, due to the intrinsic dispersions of the meta-atoms. Such phase distortions are responsible for the appearances of diffractions to the *wrong* channels, which in turn, decrease the anomalous-mode conversion efficiency from 93.5% to 54.8% and 62.9%, respectively.

### Designing tunable meta-atoms to compensate dispersions

**Figure 3:**
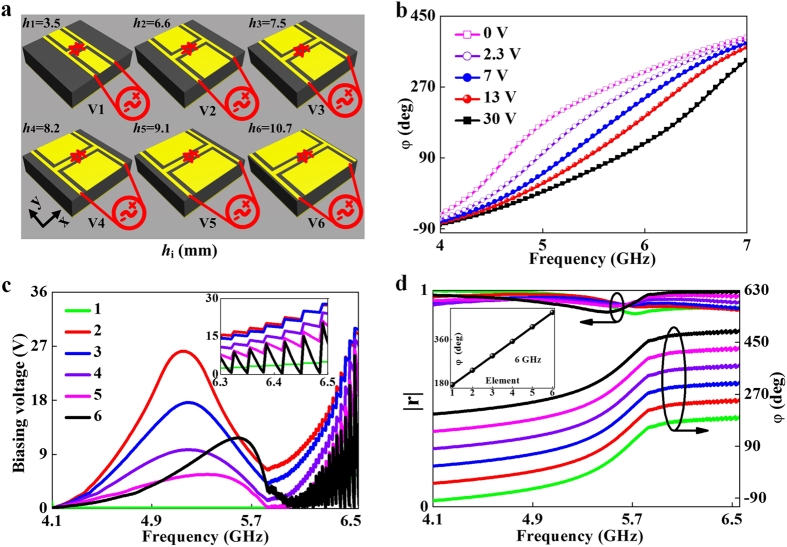
Designs and tunabilities of tunable meta-atoms. (**a**) Geometries of six tunable meta-atoms, each with a varactor diode loaded to connect the broken central line of the “H”. (**b**) Calculated reflection-phase spectra of a metasurface consisting of a periodic array of the second meta-atoms at different biasing voltages imparted on the diodes. (**c**) Retrieved values of the requested voltages imparted on six meta-atoms to maintain the linear phase distributions at every frequency within the band 4.1–6.6 GHz. Inset shows a zoom-in view of the retrieved voltages at frequencies from 6.3 to 6.5 GHz. (**d**) FDTD calculated reflection amplitudes and phases of six meta-atoms biased by the corresponding voltages as shown in (c). Inset plots the phase profile of the tunable metasurface at 6 GHz.

We describe our strategy to design tunable meta-atoms to overcome the dispersion-induced phase distortions. As shown in Fig. 3a, our tunable meta-atoms are topologically equivalent to the passive one (Fig. 2a), only with the central bars of the “H” structures broken and then connected by varactor diodes (SMV1430-079LF, Skyworks Solutions Inc.^56^), which are, in turn, *independently* controlled by six external voltages *V*_*i*_(*i* = 1, …, 6). Since the lumped-capacitance *C*_t_ of the diode depends sensitively on the voltage applied through it (see Supplementary Information), the resonance frequencies of our meta-atoms can be controlled by these external voltages. As a result, the reflection-phase spectra of six meta-atoms can be efficiently and *independently* controlled by *V*_*i*_. Figure 3b depicts how the phase of the second meta-atom *φ*_2_(*f*) varies against *V*_2_. As *V*_2_ increases from 0 to 30 V, the lumped capacitance of the diode decreases from 1.2 to 0.3 pF, which shifts the relevant resonance from 4.7 to 6.6 GHz and thus generates dramatic modulations on *φ*_2_(*f*) (see Fig. 2b). Such modulation strongly depends on the structural details of the meta-atom. Detailed *φ*_*i*_(*f*) ∼ *V*_*i*_ relationships for other five meta-atoms are presented in Supplementary Information.

With all 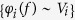 relationships known, we can retrieve the voltage combinations 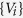 imparted on six meta-atoms at every frequency (see Supplementary Information), which can yield a *perfect* linear phase profile for the metasurface constructed by these elements. We note that the solutions of 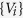 may not be unique, since what we required is merely that the phase *differences* between adjacent elements are a constant (i.e.,*φ*_*i*+1_−*φ*_*i*_ = π/3, *i* = 1, …, 5). Figure 3c depicts the retrieved 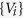 varying against frequency, which is one particular set of solutions that we found by restricting on moderate values of voltages. We note that large fluctuations exist in *V*_6_ at high frequencies. This is because the resonance frequency of the sixth meta-atom is too low, and thus the meta-atom becomes quite *inactive* with respect to external control at high frequencies. To validate our solution, we plot in Fig. 3d the spectra of reflection amplitudes and phases for six meta-atoms controlled by the external voltages given in Fig. 3c. Perfect linear phase gradient is found for every frequency within the band 4.1–6.6 GHz (see inset for the phase profile at 6 GHz), with nearly uniform reflection amplitudes (|r| > 0.9). This is quite unusual since the original resonances of these meta-atoms are completely different. Yet, under appropriate biasing voltages, we can make their phase differences keeping constant within a very broad band, overcoming the intrinsic *chromatic aberrations* in passive meta-surfaces. In principle, one can use our strategy to make a meta-atom to exhibit arbitrary reflection phase *φ*, and thus the working bandwidth can be made as large as possible. However, this is practically impossible since any diode exhibits a limited tunable range and also the external voltage cannot be made too large. These factors collectively set an upper limit on the working bandwidth of a tunable metasurface. However, we emphasize here that within such a working bandwidth the tunable metasurface exhibits an *ideal* performance without *chromatic aberrations*. Therefore, our bandwidth is intrinsically different from conventional working bandwidth for a wideband passive device^11^,^12^,^13^ in which the device’s performance gets deteriorated at frequencies deviating from the central target one.

### Far-field characterizations on the tunable metasurface: Aberration corrections

**Figure 4:**
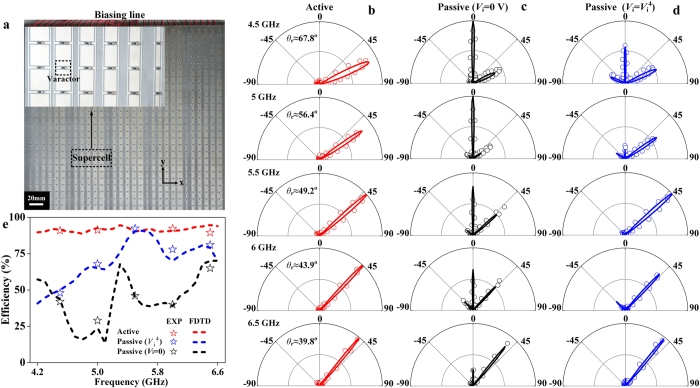
Far-field characterizations on the tunable metasurface. (**a**) Photograph of the fabricated tunable metasurface, with inset depicting a supercell of the sample. Measured (symbols) and FDTD-simulated (line) scattering patterns of the tunable metasurface at 5 frequencies, when the sample is biased (**b**) at the required voltage combination corresponding to each frequency, (**c**) at 0 V voltage, and (**d**) at the fixed voltage combination (denoted by 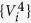) corresponding to the frequency 5.5 GHz. (**e**) Frequency-dependent anomalous-reflection efficiency of our device for the cases studied in (**b**) (red), (**c**) (blue), and (**d**) (black), obtained by analyzing the measured (symbols) and FDTD simulated (lines) scattering patterns.

We fabricated a tunable metasurface according to the design (Fig. 3a) and experimentally characterized its wave-manipulation performances. As shown in Fig. 4a, the fabricated sample contains 30 × 30 meta-atoms, with a total size of 360 × 360 mm^2^. Since the phase gradient is along the *x* direction, the meta-atoms in each vertical column are identical. Considering further the super-periodicity, we only need six independent constant-voltage sources to bias those diodes belonging to the same type of meta-atoms. According to ref. 2, we understand that our gradient metasurface functions as a SW convertor at frequencies below *f*_c_ = 4.167 GHz when the phase gradient is larger than the free-space wave-vector, while it is an anomalous reflector at frequencies above *f*_c_. We first focus on the frequency region above *f*_c_ in this subsection. Biasing the meta-atoms at their corresponding voltages as shown in Fig. 3c, we performed both microwave experiments and FDTD simulations to study the scattering patterns of our metasurface at different frequencies (see Methods section for microwave characterization details).

Figure 4b depicts the measured scattering patterns of the tunable metasurface under normal-incidence excitations at 5 frequencies (4.5, 5.0, 5.5, 6.0, 6.5 GHz). In all the cases studied, we found that the reflection beams only contain the desired anomalous mode with diffractions to the *wrong* channels fully suppressed. The experimentally identified anomalous reflection angle *θ*_*r*_ changes from 67.8^ο^ to 39.9^ο^ as frequency *f* increases from 4.5 GHz to 6.5 GHz, agreeing well with the generalized Snell’s law: 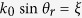 with 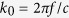 being the wave vector in free space and *c* the speed of light^1^ (see Fig. S7 in Supplementary Information). Since the phase gradient 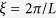 (*L* is the super periodicity) of our device is frequency independent, *θ*_*r*_ must be a decreasing function of *f*, which is verified experimentally (Fig. 4). We note that the anomalous-reflection beam becomes narrowed as *f* increases (Fig. 4b). This is again quite physical, since our metasurface becomes *effectively* enlarged in size (in term of wavelength) at higher frequencies, although its physical size remains unchanged. FDTD simulations match very well with the experimental data.

Figure 4b already indicates that our tunable metasurface exhibits nearly ideal functionalities, much more superior that its passive counterpart (see Fig. 2). Obviously, such improvement comes from the perfect linear phase gradient enabled by external controls. To further highlight the importance of the tunable phase control, we repeated all characterizations on the same device which is now under zero-voltage biasing. It is not surprising to see that the device does not show single-mode anomalous reflections at those frequencies selected (Fig. 4c). Instead, diffractions to the wrong channels are significant, similar to the passive device (Fig. 2). We note that zero-voltage-biased metasurface is not an ideal candidate to mimic a passive metasurface, since at this biasing condition the metasurface does not exhibit a good linear phase distribution at *any* frequency. Therefore, we further study the scattering properties of the same device under the biasing voltages designed for 5.5 GHz. Such a device can better mimic a passive system, since the effective lumped parameters of the diodes do not change with frequency. Figure 4d shows the measured/simulated reflection spectra of the device at the same 5 frequencies. As expected, the device works with ideal functionality only at the frequency 5.5 GHz, but the performance gets deteriorated significantly at other frequencies.

Such comparisons highlighted the key advantage of our tunable scheme — our device can work with the best functionality at any frequency within a broad band. Figure 4e compares the working efficiencies of three devices studied in Fig. 4b–d as functions of frequency. Whereas our tunable device (under appropriate biasing voltages) can always exhibit high anomalous-reflection efficiency (~90%) within 4.2–6.6 GHz, the fixed-voltage-biased device only works for a single frequency similar to a passive device, and the unbiased device exhibits poor efficiencies for all frequencies within the band.

### Near-field characterizations on the tunable metasurface: Dynamical functionality switching

We now focus on another frequency domain (*f* < *f*_*c*_), and demonstrate a further important application of our tunable scheme, that is, dynamical functionality switching. The working principle can be understood as follows. At a frequency *f* < *f*_*c*_, our gradient metasurface can function as a propagating wave (PW) to SW convertor when its phase profile *φ*(*x*) exhibits a perfect linear relationship, but becomes a normal reflector when *φ* remains nearly a constant within a supercell. Therefore, engineering *φ*(*x*) in a designed manner can create two “states” between which the metasurface can switch.

**Figure 5:**
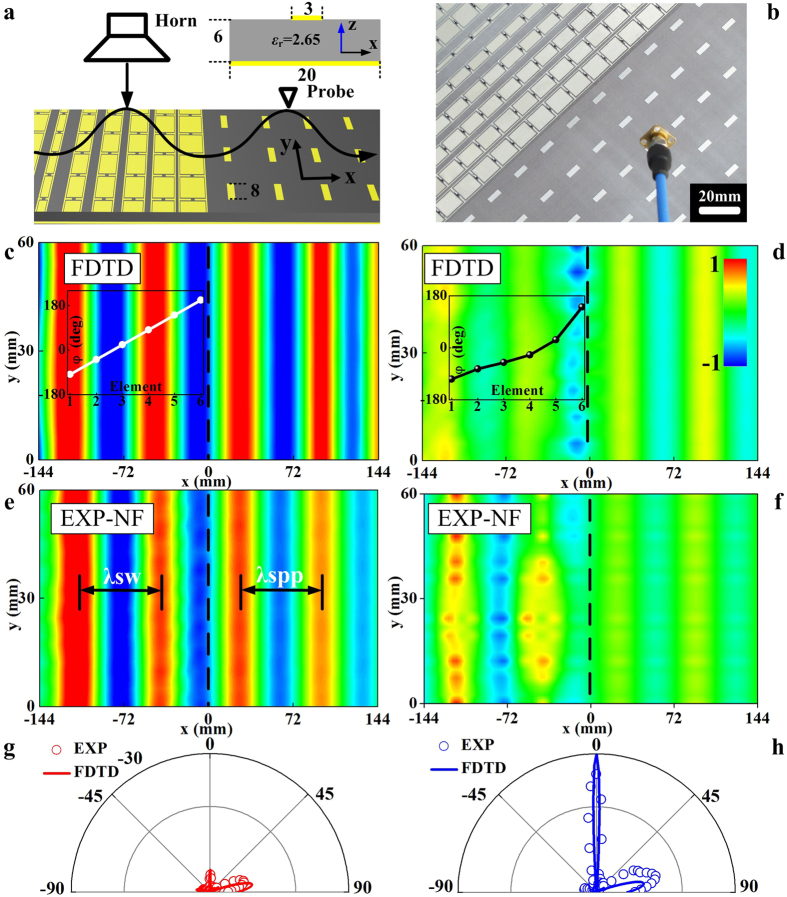
Near-field characterizations on the tunable metasurface: Dynamical functionality switching. (**a**) Schematics of the PW-SW conversion setup. Inset shows a unit cell of the mushroom structure, consisting of a metal bar (sized 3 mm × 8 mm) coupled with a continuous metal plate through a 6 mm – thick dielectric spacer (*ε*_*r*_ = 2.65). (**b**) Picture of the fabricated sample and the near-field probe. (**c**,**d**) FDTD simulated and (**e**,**f**) measured Re(*E*_z_) distributions under (**c**,**e**) the voltage combination corresponding to frequency 4.1 GHz as shown in Fig. 3(c) and (d,f) *V*_i_ = 30 V for all elements. Insets in (c) and (d) depict the phase profiles under the specified external voltages. All fields in (c–f) share the same color bar shown in (d). FDTD simulated and measured far-field scattering patterns of the metasurface at 4.1 GHz under (**g**) the voltage combination corresponding to frequency 4.1 GHz and (**h**) *V*_i_ = 30 V for all elements.

We experimentally demonstrated this idea at 4.1 GHz. Following ref. 2, we first design and fabricate a mushroom structure (see inset for its unit-cell geometry) supporting spoof surface plasmon polariton (SPP) propagations at this frequency, and then place it at the right-hand side of the gradient metasurface (Fig. 5a). Such a device can guide out the SW generated on the metasurface, when the latter is shined by a normally incident PW. The real experimental sample and setup are shown in Fig. 5b. In our experiment, shining the metasurface *alone* by an *x*-polarized PW, we employed a monopole antenna to measure the distribution of local Re(*E*_*z*_) fields generated on both the metasurface and the mushroom surface. To ensure a high conversion efficiency, the mushroom structure is carefully designed such that the wave vector of its spoof SPP is *k*_spp_ ≈ 1.02*k*_0_ at 4.1 GHz (see Supplementary InformationM), which is matched to the wavevector of the “driven” SW generated on the metasurface.

We performed near-field (NF) measurements in two cases where the meta-atoms are biased at two different voltage combinations. In the “On”-state, the biasing voltages are chosen from Fig. 4c so that *φ*(*x*) exhibits perfect linear relationship (inset to Fig. 5c). In the “Off”-state where all voltages are set as 30 V, however, the *φ*(*x*) relation deviates significantly from a linear one and *φ* remains nearly unchanged in the large area of a supercell (inset to Fig. 5d). As expected, when the metasurface is set at the “On”-state, we do observe very strong spoof SPP signals and the simulated/measured NF patterns (Fig. 5c and e) exhibit a very well-defined *k*_spp_ ≈ 1.02*k*_0_, indicating that our device is a very efficient SW convertor. On the other hand, when the metasurface is switched to the “Off”-state, we only detected negligible SPP signals on the mushroom structure with an On/Off power ratio 22.1, implying that the device now ceases to behave as a SW convertor (Fig. 5d and f). To further differentiate the functionalities of our device at two states, we performed far-field (FF) experiments to measure the scattering patterns (Fig. 5g and h) of our device at two different states. Combining experimental data from NF and FF measurements, we conclude that our device is mostly a SW convertor in the “On”-state, but dynamically changes to a conventional reflector in the “Off”-state.

## Discussion

To summarize, we established a tunable scheme to overcome the dispersion-induced issues in microwave metasurfaces. Adding tunable elements to our meta-atoms, we can precisely control the phase response of each meta-atom via external knobs, thereby to rectify the inevitable phase distortions at arbitrary frequencies. Two distinct effects were experimentally demonstrated: (1) We can realize tunable metasurfaces exhibiting the best functionalities at every frequency within a broad band, overcoming the dispersion-induced phase distortions intrinsic to all passive devices; (2) We can dynamically switch the functionalities of metasurfaces via changing the external controls. Our findings pave the road to realize tunable meta-devices achieving dispersion-compensated and/or dynamically switchable high performances. Extensions to high-frequency domains are even more interesting, based on modern technologies such as optical pumping on semiconductors^44^ and gate-tunable dielectric materials^57^.

## Methods

### Simulations

All full-wave FDTD simulations were performed with a numerical solver (CONCERTO 7.0, Vector Fields Limited, UK (2008)). In the FDTD simulations, metals were treated as perfect electric conductors and the varactor was modeled by a series of RLC lumped elements containing an inductor (*L*_s_ = 0.7 nH), a resistor (*R*_t_ = 1.5 Ω) and a capacitor with *C*_t_ varying inside [0.3 pF, 1.2 pF] depending on the externally applied voltage (see Fig. S1b in Supplementary Information). In the simulation domains occupied by one of these lumped elements, the 4^th^ Maxwell’s equation is modified as 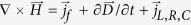, where, other than conventional free and displacement currents 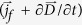, an additional term 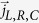 is added to describe the responses of the lumped elements. These currents are determined by the voltages *U* applied across the lumped elements through the circuit equations 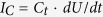, 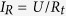 and 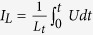, which are in turn, related back to the electric fields defined in the corresponding meshes. By doing so, we can self-consistently incorporate the responses of the lumped elements in the FDTD simulations. In the calculations of reflection magnitudes/phases of meta-atoms, we studied a unit cell containing a single meta-atom with periodic conditions applied at its four boundaries and a floquet port assigned at a distance 15 mm away from the *xy*-plane where the meta-atom is placed. In the far-field calculations, we chose to study a line of meta-atoms belonging to five supercells arranged in the *x* direction, with open boundary conditions set as its two ends and periodic boundary conditions set at its two boundaries along the *y* direction. Similarly, in near-field calculations, the studied system contains five/three super cells along the *y*/*x* direction, again with periodic/open boundary conditions imposed at the boundaries. In all cases, the metasurface/meta-atom is shined by a normally incident *x*-polarized plane wave. The varactor SMV1430-079LF can only work well for frequencies below 10 GHz. Above 10 GHz, unreliable capacitance/inductance values and large self-resonant loss will be induced in the varactor, which is undesirable for our applications. The higher frequency micro-electromechanical system diode affords an extremely low loss strategy and thus would benefit much the high absolute efficiency.

### Sample fabrications

The active metasurfaces were fabricated using printed-circuit-board technique with all varactor diodes attached to the top metallic microstructure using surface-mount technology. To guarantee perfect electrical connections with correct electrodes, all varactors are checked by amultimeter of VICTOR VC9807A+. To control the capacitances of the varactor diodes, we connected constant-voltage sources (GPD4303S, GWINSTEK company, Taiwan) to their two ends by two thin wires. To suppress the leakage of microwave signals and thus enable a robust and reliable performance, the top and bottom horizontal bars functioning as zero- and reverse-biased line, respectively are engineered thinner than the perpendicular bar to provide a high reactance.

### Microwave characterizations

In the far-field experiments, shining the metasurface by a microwave beam (with spot size ~250 mm at 6 GHz (E plane)) emitted from a linear-polarization horn antenna placed 1.2 m away, we measured the scattered-field intensity with another identical horn antenna, which can be freely rotated (with a step of 3°) on a circular track with 1.2 m radius. All scattered power was recorded by a vector network analyzer (Agilent E8362C PNA) and was normalized against a reference signal *P*_0_, obtained by measuring the specularly reflected signal with the metasurface replaced by a metallic plate with the same size. In measuring *P*_0_, two horn antennas were placed at the same side of the sample with a 10° angle separation. In the near-field experiments, we placed a 15 mm-long monopole antenna perpendicular to the metasurface, moving at a plane 3 mm above the metasurface, to measure the local *E*_*z*_ field (with both amplitude and phase). Absorbing materials are placed at the right side of the mushroom structure to prevent any reflected signals.

## Additional Information

**How to cite this article**: Xu, H.-X. *et al*. Tunable microwave metasurfaces for high-performance operations: dispersion compensation and dynamical switch. *Sci. Rep.* **6**, 38255; doi: 10.1038/srep38255 (2016).

**Publisher’s note:** Springer Nature remains neutral with regard to jurisdictional claims in published maps and institutional affiliations.

## Supplementary Material

Supplementary Information

## Figures and Tables

**Figure 1:**
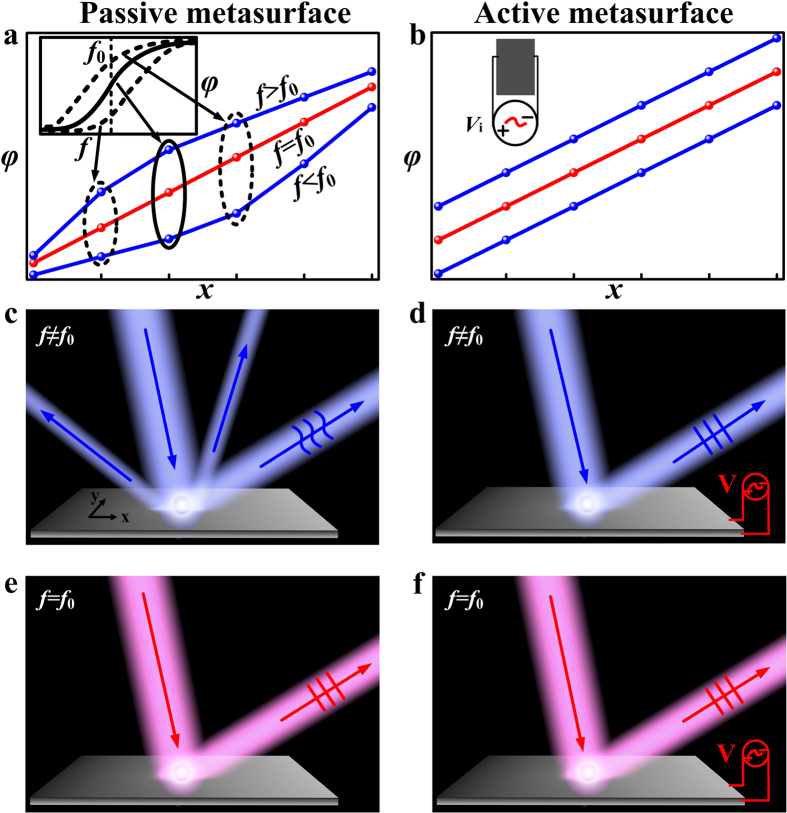
Tunable scheme to overcome the phase distortions in passive metasurfaces. Typical phase responses of meta-atoms within a supercell in a (**a**) passive and (**b**) a tunable gradient metasurface, at working frequency *f* = *f*_0_ and non-working frequencies *f* ≠ *f*_0_. The nonlinear phase distributions at non-working frequencies in the passive device, caused by frequency dispersions of passive meta-atoms (see inset to Fig. 1a), can be rectified by active external controls in the tunable device. Typical scattering patterns of (**c**,**e**) passive or (**d**,**f**) tunable metasurfaces at (**c**,**d**) off-working frequencies and (**e**,**f**) working frequency.
