# Supplementary material for: Tunable microwave metasurfaces for high-performance operations: dispersion compensation and dynamical switch
Source: Sci Rep. 2016 Nov 30;6:38255. doi: 10.1038/srep38255 (PMC5128812; doi:10.1038/srep38255)
Supplement: Supplementary Information [file srep38255-s1.doc]

**Supplementary Information**

# Tunable microwave metasurfaces for high-performance operations: dispersion compensation and dynamical switch

He-Xiu Xu^1,2^, Shiwei Tang^1,5^, Shaojie Ma^1^, Weijie Luo^1^, Tong Cai^1,2^, Shulin Sun^3^, Qiong He^1,4^*, Lei Zhou^1,4^*

^1^*State Key Laboratory of Surface Physics, Key Laboratory of Micro and Nano Photonic Structures (Ministry of Education) and Physics Department, Fudan University, Shanghai 200433, China*

^2^*Air and Missile Defense College, Air force Engineering University, Xi'an, 710051, China*

^3^ *Shanghai Engineering Research Center of Ultra-Precision Optical Manufacturing, Green Photonics and Department of Optical Science and Engineering, Fudan University, Shanghai 200433, China*

^4^*Collaborative Innovation Center of Advanced Microstructures, Fudan University,*

*Shanghai 200433, China*

^5^*Department of Physics, Faculty of Science, Ningbo University, Ningbo 315211, China*

*Corresponding Authors: Lei Zhou, E-mail: phzhou@fudan.edu.cn; Qiong He, E-mail: qionghe@fudan.edu.cn.

[1. Additional information on how to control the two resonant modes in our meta-atoms 2](#__RefHeading___Toc442522019)

[2. Additional results for the passive metasurface studied in Fig. 2 5](#__RefHeading___Toc442522020)

[3. CAD approach to search for the solutions of biasing voltages 6](#__RefHeading___Toc442522021)

[4. Additional results for the active metasurface studied in Fig. 4 7](#__RefHeading___Toc442522022)

[5. Dispersion curve of the spoof SPP on the mushroom structure 10](#__RefHeading___Toc442522023)

[6. Pictures of the experimental setup 10](#__RefHeading___Toc442522026)

# Additional information on how to control the two resonant modes in our meta-atoms

Since our meta-atom is much smaller than the wavelength of interest, its resonant behavior and tunability can be understood from a lumped LC circuit. To offer a guideline for the metasurface design, here we establish the equivalent circuit model (CM) for our structure (Fig. S1a). The two magnetic resonances (at *f*_1_ and *f*_2_) can be modeled by two series resonant tanks formed by *L*_1_, *C*_1_ and *R*_1_, and *L*_2_, *C*_2_ and *R*_2_, respectively. The back metallic layer was represented by the ground, whereas the transmission through the dielectric substrate (with impedance *Z*_o_ and thickness *h*) was modeled by a transmission line (TL) with equivalent impedance *Z*_c_ and electrical length *h*_o_. In Fig. S1b, *R*_t_, *L*_t_ and *C*_t_ in the detection model represent the total resistance, total inductance and total capacitance of the whole system, which have included the contributions from the junction resistance *R*_s_, the lead inductance *L*_s_ and the package capacitance *C*_s_ in the spice model. In our design, *C*_s_ is negligible compared to the junction capacitance *C*_j_ of the varactor SMV1430-079LF, yielding *C*_t_≈*C*_j_. In full-wave FDTD simulations, the varactor is replaced by a series resonant tank with *L*_s_=*L*_t_=0.7 nH, *R*_s_=*R*_t_=1.5 Ω and 0.3 pF≤*C*_t_≤1.2 pF. Using Agilent’s Advanced Design System (ADS), we obtained the $C_{t}\sim V$ curve for our varactor (inset to Fig. S1b), which is extremely helpful for our design. As is shown, *C*_t_ decreases from 1.2 to 0.3 pF when *V* increases from 0 to 30 V. Moreover, *C*_t_ changes sharply when *V* is small and saturates at a certain value when *V* approaches 15 V. Therefore, one can choose a small *V* to obtain a sharp tunability. The electric-current distributions associated with the same resonant mode remain the same in the passive (Fig. S1c) and active (Fig. S1d) meta-atoms, although the resonant frequencies are at different positions. The induced currents are mainly around the “H” in the first mode (*f*_1_), but are mainly on the patch surface in the second one (*f*_2_), consistent with the picture set up in Fig. 2(b) of the main text. However, we found strong couplings between the patch and the “H” at the second mode (*f*_2_), evidenced by strong overlapping of electric fields belonging to two different structures. Such coupling blue-shifted *f*_2_ in the composite resonator relative to the patch-only case. However, *f*_1_ was hardly affected by the existence of the patch since such coupling is remarkably weak at *f*_1_. Based on Figs. S1c and S1d, we got *L*_1_≈*L*_w_+*L*_t_, *L*_2_≈*L*_p_/2, *C*_1_=*C*_f_**C*_t_/(*C*_f_+*C*_t_), and *C*_2_≈2(*C*_p_+*C*_c_), where *L*_w_ is contributed by the perpendicular bar, *C*_f_ models the fringing capacitor formed between parallel horizontal bars of two adjacent elements, *L*_p_ and *C*_p_ are the inductor and capacitor of the patch, respectively. The losses of above two tanks are modeled by *R*_1_ and *R*_2_, respectively. TL theory tells us that and , and thus the response of our active meta-atom (at an arbitrary frequency) can be efficiently tuned by engineering these parameters (i.e., $L_{1},C_{1},L_{2},C_{2}$ *etc.*).

**
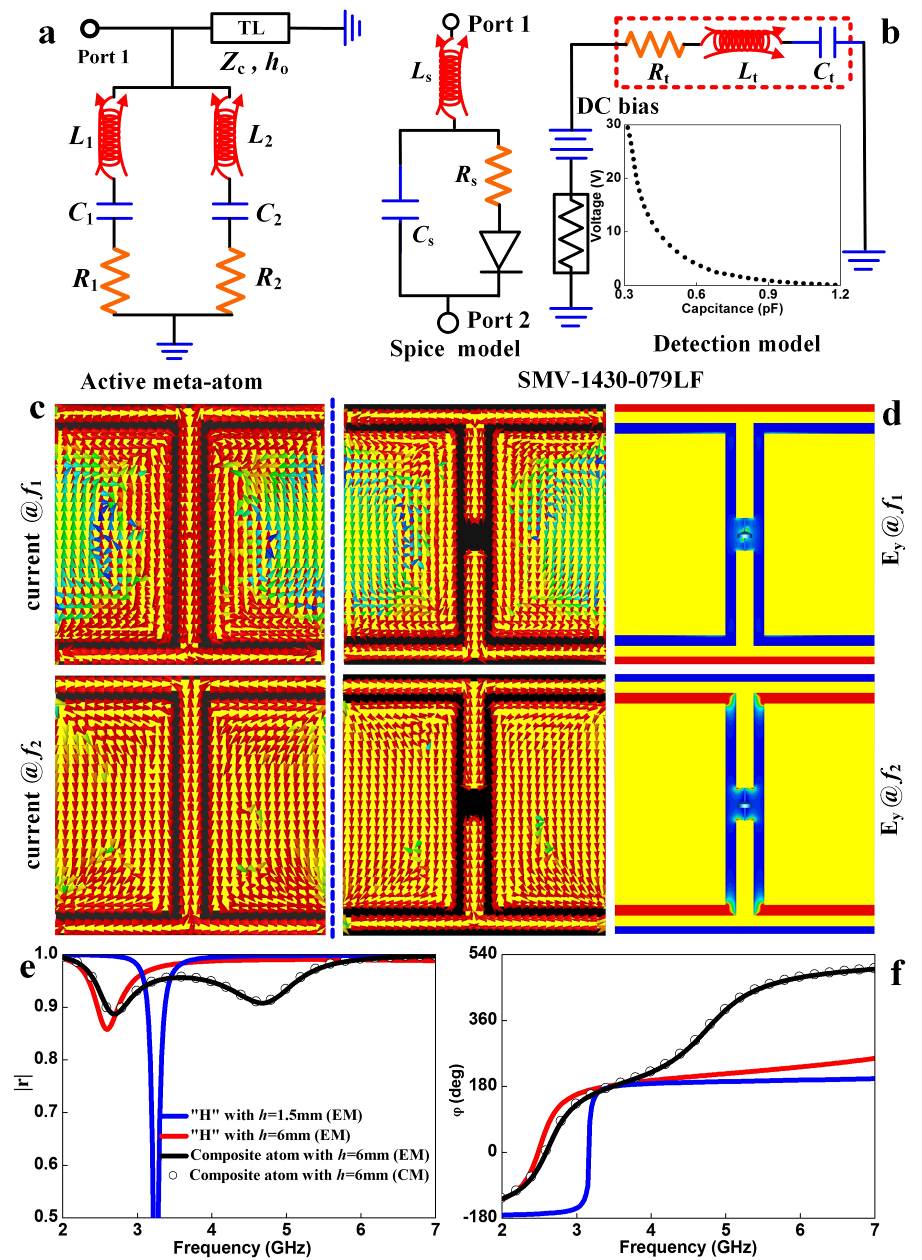
**

**Figure S1 | Analyzing the two resonant modes with FDTD and Circuit Model studies.** The equivalent CM for (**a**) active/passive meta-atom and (**b**) varactor SMV1430-079LF. Inset shows the C-V curve of the varactor. Field/current distributions for the (**c**) passive and (**d**) active meta-atoms, respectively. CM and EM simulated reflection (**e**) amplitude (|r|) and (**f**) phase (φ) of the active meta-atom and the “H”-shaped meta-atom with different *h*. Other geometrical parameters are *p*_x_=*p*_y_=12 mm, *w*_1_=0.8 mm, *w*_2_=0.5 mm, *w*_3_=5.1 mm, *d*_1_=0.25 mm, *d*_2_=0.5 mm, *d*_3_=10 mm and *h*_i_=10.5 mm. The circuit parameters are retrieved as *L*_1_=18.76 nH, *C*_1_=0.111 pF, *L*_2_=0.059 nH, *C*_2_=0.196 pF, *R*_1_=8.37 Ω, *R*_2_=0.114 Ω, *Z*_c_=204.9 Ω and *h*_o_=58.9^o^.

When discussing the two modes in the main text, we only depicted the reflection coefficients of a *passive* meta-atom in Fig. 2b. Here, we compared in Figs. S1e and S1f the spectra of reflection amplitude and phase of a typical *active* meta-atom with $h=6\text{ mm}$ and biasing voltage of *V*_i_=0 V, obtained by FDTD simulations (black lines) and CM analyses (open circles). Reasonable agreement between the two results validated our CM. Compared to its passive counterpart (see Fig. 2 in the main text), our active meta-atom exhibits similar EM responses but with an enhanced Q factor due to the additional LC elements contributed by the active diode. Therefore, cascading two resonances in our design is crucial to enlarge the phase/frequency tuning range. In addition, increasing the spacer thickness (*h*) is another approach to decrease the Q factor (see Fig. S1e-f).


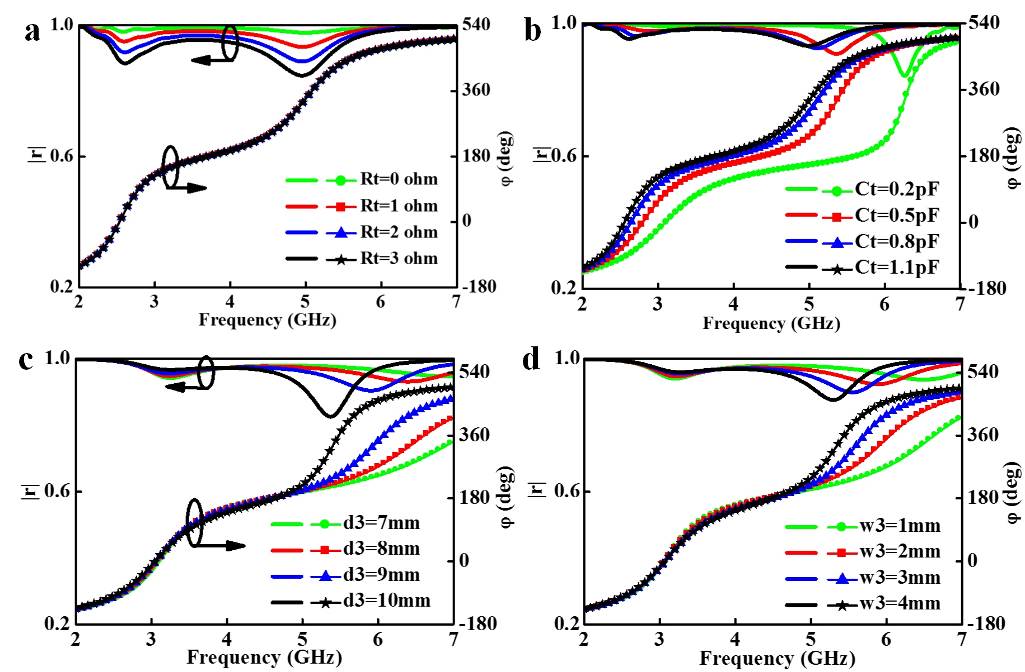


**Figure S2 | EM properties of the active meta-atom depending on different parameters.** FDTD calculated spectra of reflection amplitude $|r|$ and phase for an active meta-atom with different (a) $R_{t}$ (*d*_3_=10.16 mm, *w*_3_=5.1 mm and *C*_t_=1.2 pF fixed), (b) *C*_t_ (*d*_3_=10.16 mm, *w*_3_=5.1 mm and *R*_t_=1 Ω fixed), (c) *d*_3_ (*w*_3_=5.1 mm, *R*_t_=1 Ω and *C*_t_=0.31 pF fixed), and (d) *w*_3_ (*d*_3_=10 mm, *R*_t_=1 Ω and *C*_t_=0.31 pF fixed). Other geometrical parameters are *p*_x_=*p*_y_=12 mm, *w*_1_=0.8 mm, *w*_2_=0.5 mm, *d*_1_=0.25 mm and *d*_2_=0.5 mm.

To understand the roles of different parameters to control the EM responses of our active meta-atoms, we performed extensive simulations by varying the circuit parameters *R*_t_, *C*_t_ and the patch parameters *w*_3_, *d*_3_ while maintaining other parameters unchanged. Figure S2a shows that increasing *R*_t_ can increase the absorption dip but has nearly no effect on the phase spectra. Meanwhile, Fig. S2b shows that both *f*_1_ and *f*_2_ undergo a red-shift when *C*_t_ increases from 0.2 to 1.1 pF. The capacitive coupling between the patch and the “H” enables such a simultaneous control. Most importantly, the reflection phase can be progressively tuned to cover a 270^o^ phase modulation within 2~7 GHz. Finally, Figs. S2c and S2d show that varying *w*_3_ or *d*_3_ can only modulate the resonance at *f*_2_ or *f*_1_, consistent with Fig. 2(b) in the main text. To sum up, we note that the external biasing (Fig. S2b) provides a further way to control the reflection phase of our meta-atom in addition to the geometrical tuning (Fig. S2c-d), and the possibility to separately control two different resonances helps us achieve arbitrary values for $f_{1}/f_{2}$.

# Additional results for the passive metasurface studied in Fig. 2

In Fig. 2 of the main text, we plotted the simulated scattering patterns and the working efficiencies of the passive metasurface. Here, we provide more details on the design process and additional results on the properties of the passive metasurface.

Figure S3a-3b depict how the reflection amplitude/phase of our passive meta-atom (see Fig. 2a in the main text for its geometry) varies against parameter *h*_i_ and the target frequency. Obviously, $|r|$ is always near 1 which we do not need to worry about (Fig. S3a), but strongly depends on both *h*_i_ and frequency (Fig. S3b), since the resonant frequency of the meta-atom sensitively depends on the value of *h*_i_. Set the working frequency at *f*_0_ = 5.7 GHz, a careful parameter searching on Fig. S3b helps us obtain the final design of the passive gradient metasurface (see inset to Fig. 2a). To justify our design presented in the main text, we plotted in Fig. S3c the reflection amplitude/phase spectra of our six meta-atoms, from which we find a perfect linear phase gradient of π/3 (109.3, 169.2°, 229.4°, 289.6°, 349.5° and 409.6°) at 5.7 GHz, with all reflection amplitudes larger than 0.97. However, such a linear phase gradient cannot be strictly held at other frequencies due to different frequency dispersions of such dual-mode meta-atoms. In particular, the phase gradient decreases to zero at the boundaries of the working band (see Fig. S3c) where all resonances die off. Figure S3d shows the calculated scattered-field intensity versus frequency and receiving angle $\theta_{r}$, as our metasurface is illuminated by a normally incident *x*-polarized wave. As expected, we observed a high anomalous reflection and suppressed m=0 and m=-1 diffractions only around 5.7 GHz, where a linear phase gradient is strictly held. At other frequencies, diffractions to the “wrong” channels are significant which decrease the desired working efficiency (Fig. S3d). The two additional side peaks at 5 and 6.7 GHz in Fig. 2d are caused by the enhanced linearity (see inset to Fig. S3c), accidentally existing for such dual-mode meta-atoms.


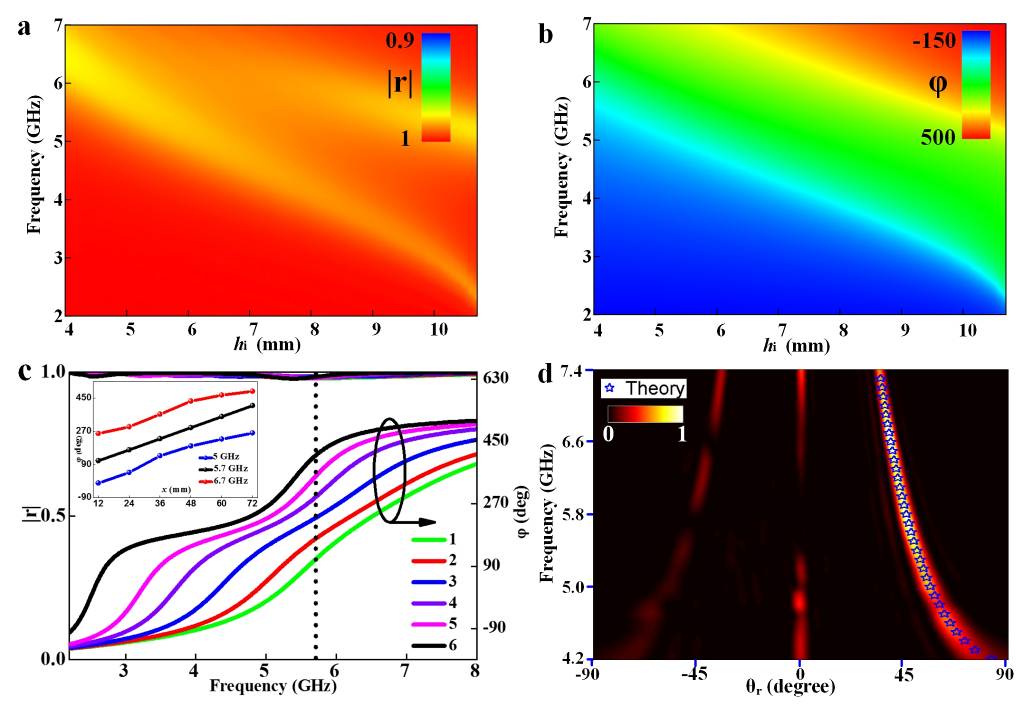


**Figure S3 |** **Additional FDTD results on the passive meta-atoms and metasurface studied in Fig. 2.** (**a**) Reflection amplitude ($|r|$) and (**b**) phase () of a basic passive meta-atom (see inset to Fig. 2(a)) versus frequency and the parameter *h*_i_. (**c**) Spectra of $|r|$ and for six passive meta-atoms (see Fig. 2(a) for their geometrical parameters), with inset showing the profiles of the passive metasurface at 5, 5.7 and 6.7 GHz. (**d**) 2D contour for the far-field reflected power versus frequency and reflection angle for the passive metasurface. Blue stars are the results calculated with the generalized Snell’s law. The scattered field intensity P(*θ*_r_, λ) was normalized against *P*_0_, which is obtained with the metasurface replaced by a PEC plate with the same dimensions.

# CAD approach to search for the solutions of biasing voltages

We established a CAD (computer-aided design) method to search for the required biasing voltages $\{V_{i}\}$ imparted on the meta-atoms to yield the desired phase distributions of the metasurfaces. The design procedure consists of the following four steps (Fig. S4). First, we determine the periodicity *p*_i_ of meta-atoms, phase gradient *φ*_0_ (the number of elements *N* used in a super cell) at initial frequency *f*_0_ and capacitance *C*_0_. To engineer linear/parabolic phase gradient at *f*_0_, we can readily synthesize the initial geometrical parameters of above *N* elements with the help of CM theory by performing FDTD optimizations. In this particular design, *C*_0_ is chosen as the maximum value that a varactor can afford such that the phase gradient can be restored at *f*>*f*_0_. Second, we obtain the phase responses of the above *N* elements versus frequency and capacitance *C*_t_ by FDTD parametric analyses. Then, a capacitance-phase (*C*_t_–*φ*) relation curve is obtained. Third, we achieve all possible *φ*/*C*_t_ solutions of *N* elements at each frequency according to the *C*_t_–*φ* curve by employing spline interpolating method and traversal query (a root finding algorithm). Since the *φ*/*C*_t_ solutions of *N* elements satisfying perfect phase gradient may not be unique at each frequency, we select the solution that exhibits minimum *C*_t_ span between *N* atoms to guarantee the optimum bandwidth. The root finding process will be continued at the next frequency if the optimum *C*_t_ of *N* elements all falls in the capacitance tuning range (0.3 pF<*C*_t_<1.2 pF in this particular design) at the present frequency. Otherwise, it will be terminated and the operation bandwidth of the active metasurface is determined. In the fourth step, we derive the required voltages of *N* elements at each frequency according to the capacitance-voltage (C-V) curve of the varactor. To guarantee sufficient precision, numerical interpolation is also adopted for the C-V relation with a mass of samples.


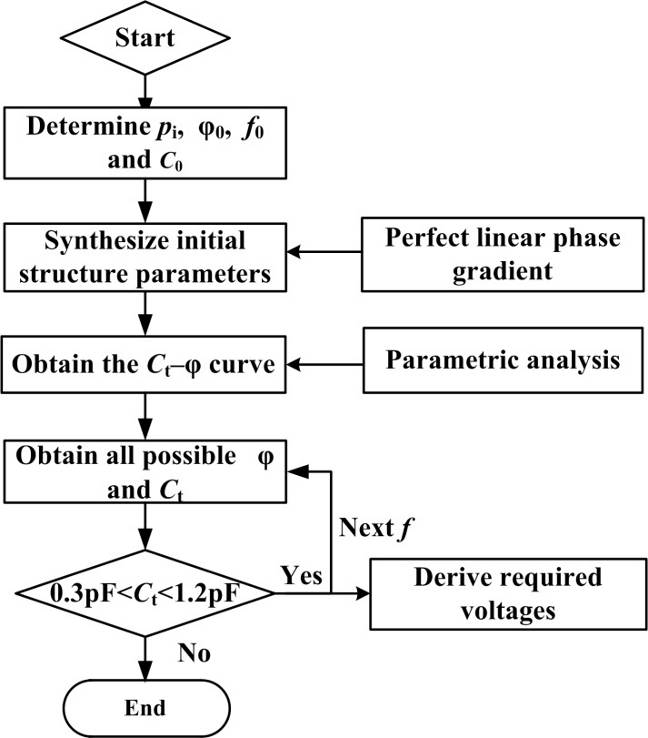


**Figure S4 | The CAD flow chart for broadband phase gradient design.**

# Additional results for the active metasurface studied in Fig. 4

To design the active metasurface studied in Fig. 4 of the main text, we first vary *h*_i_ of six meta-atoms (keeping other parameters unchanged) to ensure that the phase profile of our meta-surface is perfectly linear at an initial frequency *f*_0_=4.1 GHz under *V*_i_=0 V voltage (*C*_t_≈1.2 pF). Figure S5a-5b depict how $|r|$ and of the “active” meta-atom (with *V_i_*=0 V fixed) vary against its *h*_i_ and frequency while keeping other geometrical parameters unchanged. We note that the “active” meta-atom, when it is under fixed biasing voltage, behaves similarly to a passive one (see Fig. S3a-b), except for slightly enhanced absorption brought by the inserted active element. Figure S5a-b assist us to fix the geometries of our six meta-atoms (denoted by 1, 2, 3, 4, 5 and 6 for convenience), which exhibit *h*_i_=3.5, 6.6, 7.5, 8.2, 9.1 and 10.7 mm, respectively (all other parameters are fixed). To validate our design, we show in Fig. S5c the calculated spectra of reflection amplitudes and phases for the six active meta-atoms with zero biasing voltages. At *f*_0_=4.1 GHz, we find that the phases of these meta-atoms do exhibit a perfect linear phase gradient (see Fig. S5d), while their reflection amplitudes are all larger than 0.9 with fluctuations less than 0.1 (Fig. S5c).

We next employ the CAD design strategy (Fig. S4) to search for the voltage combinations$\{V_{i}(f)\}$to yield perfect linear phase relationships at frequencies *f*>*f*_0_. Figure S6a-f depict how the reflection phases of six meta-atoms vary against *C*_t_ and frequency. As expected, tuning each *C*_t_ (through varying the voltage $V$imparted on the very meta-atom) can significantly modulate the reflection phase of this active meta-atom (see Figs. S6a-f) within the frequency range 3.2 to 7GHz. Based on these data (Figs. S5-6) and the $C_{t}\sim V$relation (see Fig. S1b), we finally obtained the solutions$\{V_{i}(f)\}$as depicted in Fig. 3c of the main text.


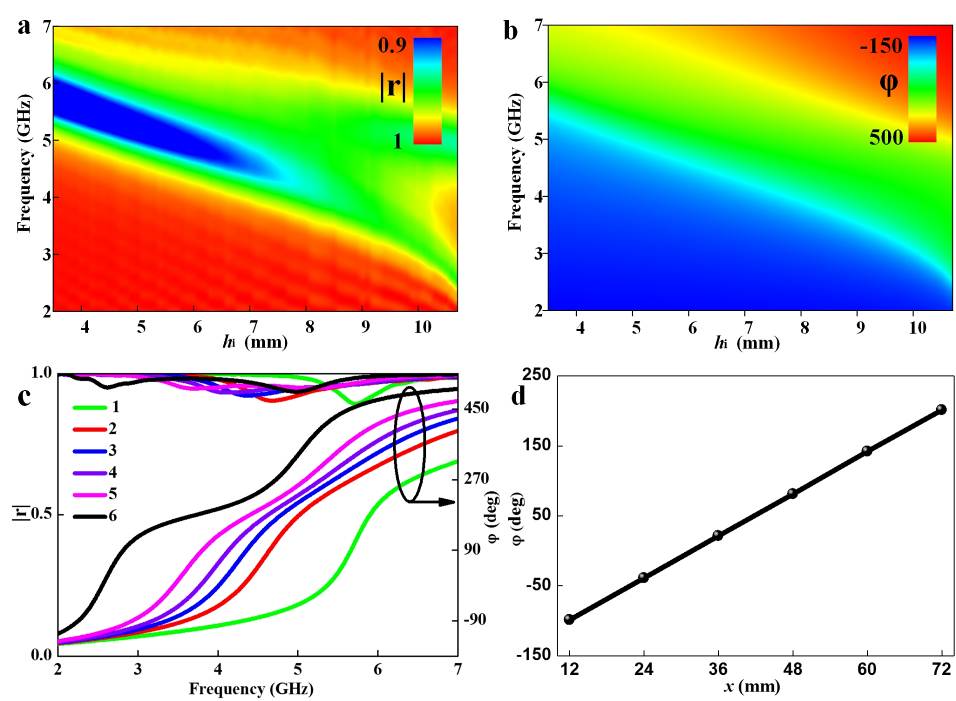


**Figure S5 | FDTD results of the active meta-atoms studied in the main text under zero biasing voltages.** Computed reflection (**a**) amplitude ($|r|$) and (**b**) phase () of an active meta-atom (under zero biasing voltages) versus frequency and its parameter *h*_i_. The super cell of the metasurface is formed by six meta-atoms with *h*_i_=3.5, 6.6, 7.5, 8.2, 9.1 and 10.7 mm, respectively. (**c**) FDTD simulated spectra of $|r|$ and of six meta-atoms under zero biasing voltages. (**d**) Phase profile of the active metasurface formed by the six meta-atoms, under zero biasing voltages at the frequency 4.1 GHz.


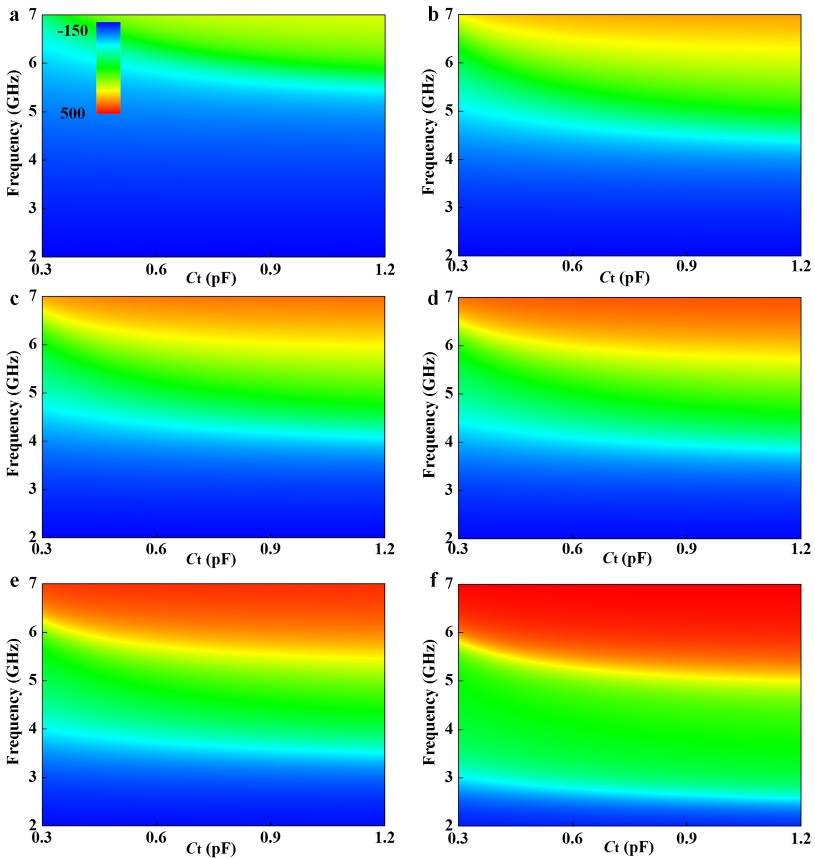


**Figure S6 | FDTD parametric analyses on six active meta-atoms.** The reflection phases versus *C*_t_ and frequency for active meta-atoms with (**a**) *h*_i_=3.5 mm, (**b**) *h*_i_=6.6 mm, (**c**) *h*_i_=7.5 mm, (**d**) *h*_i_=8.2 mm, (**e**) *h*_i_=9.1 mm and (**f**) *h*_i_=10.7 mm.

Figure S7 compares the $\theta_{r}\sim f$relations obtained by experiments, FDTD and the generalized Snell’s law, for the active meta-surface studied in the main text (Fig. 4b). Good agreements are noted among different results. Slight deviations at low frequencies are induced by the finite-size effect, since the size of our metasurface becomes *effectively* shortened in term of wavelength at low frequencies.


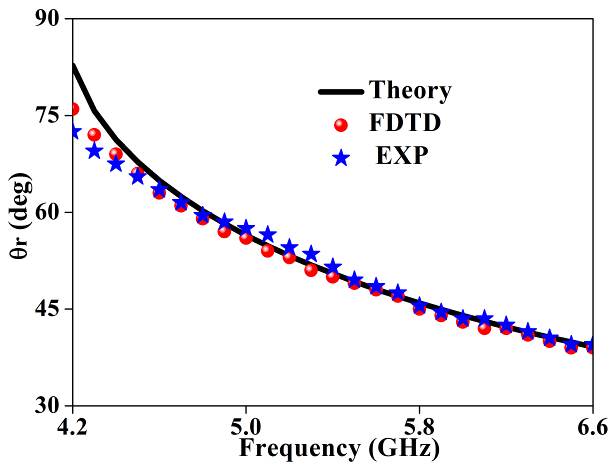


**Figure S7 | Verifications on the generalized Snlle’s Law.** $\theta_{r}\sim f$relations obtained by experiments, FDTD and the generalized Snell’s law, for the active meta-surface studied in the main text (Fig. 4b).

# Dispersion curve of the spoof SPP on the mushroom structure

To obtain a high efficiency for the SPP coupler (see Fig. 5 in the main text), the wave vector $k_{\text{SPP}}$of the spoof SPP on the mushroom structure should match with the phase gradient $\xi$ of the active metasurface. Figure S8 depicts the FDTD computed dispersion curve of spoof SPP on the mushroom structure with geometric parameters given in the inset to Fig. 5a of the main text. At 4.1GHz, we do find $k_{\text{SPP}}=1.02k_{0}=\xi$.


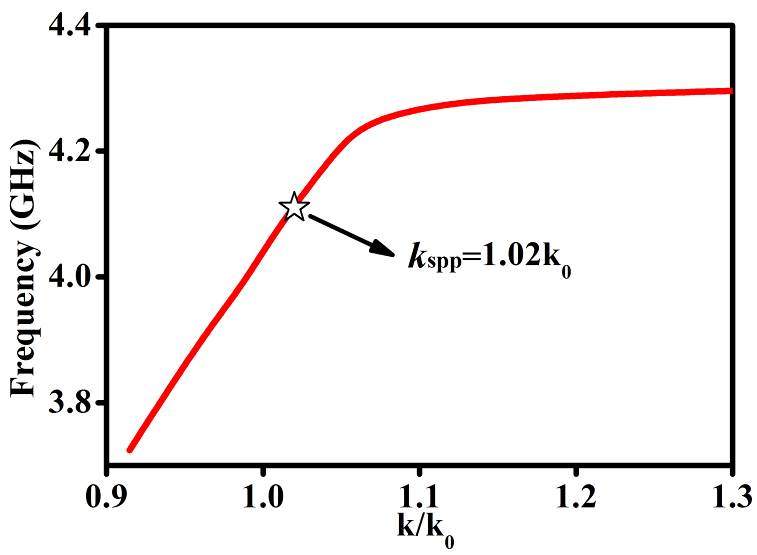


**Figure S8 | Computed dispersion curve of spoof SPP on the mushroom structure adopted in the main text.**

# Pictures of the experimental setup


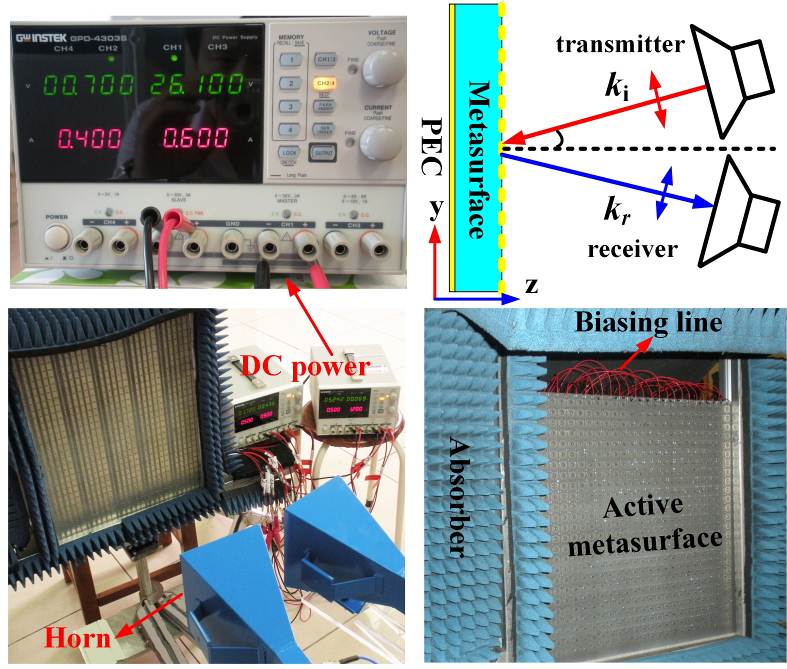


**Figure S9 | Illustration of the angle-resolved reflection measurement setup.**
